# Serological Surveillance of Rabies in Free-Range and Captive Common Vampire Bats *Desmodus rotundus*

**DOI:** 10.3389/fvets.2021.681423

**Published:** 2021-09-29

**Authors:** Jane Megid, Julio Andre Benavides Tala, Laís Dário Belaz Silva, Fernando Favian Castro Castro, Bruna Letícia Devidé Ribeiro, Camila Michele Appolinário, Iana Suly Santos Katz, Karin Corrêa Scheffer, Sandriana Ramos Silva, Adriana Ruckert Rosa, Gisely Toledo Barone, Luzia Fátima Alves Martorelli, Marilene Fernandes de Almeida

**Affiliations:** ^1^School of Veterinary Medicine and Animal Science, São Paulo State University (Unesp), Botucatu, Brazil; ^2^Centro de Investigación para la sustestabilidad, Facultad de Ciencias de la vida, Universidad Andrés Bello, Santiago, Chile; ^3^Animal Health Department, Universidad Antonio Narino Cauca Popayan, Popayán, Colômbia; ^4^Diagnostics Sector, Immunology Laboratory Pasteur Institute of São Paulo, São Paulo, Brazil; ^5^Centro de Controle de Zoonoses, Coordenadoria de vigilância em saúde, São Paulo, Brazil

**Keywords:** rabies, serology, RFFIT, vampire bats, antibodies, virus neutralizing antibodies

## Abstract

The control of vampire bat rabies (VBR) in Brazil is based on the culling of *Desmodus rotundus* and the surveillance of outbreaks caused by *D. rotundus* in cattle and humans in addition to vaccination of susceptible livestock. The detection of anti-rabies antibodies in vampire bats indicates exposure to the rabies virus, and several studies have reported an increase of these antibodies following experimental infection. However, the dynamics of anti-rabies antibodies in natural populations of *D. rotundus* remains poorly understood. In this study, we took advantage of recent outbreaks of VBR among livestock in the Sao Paulo region of Brazil to test whether seroprevalence in *D. rotundus* reflects the incidence of rabies in nearby livestock populations. Sixty-four *D. rotundus* were captured during and after outbreaks from roost located in municipalities belonging to three regions with different incidences of rabies in herbivores. Sixteen seropositive bats were then kept in captivity for up to 120 days, and their antibodies and virus levels were quantified at different time points using the rapid fluorescent focus inhibition test (RFFIT). Antibody titers were associated with the occurrence of ongoing outbreak, with a higher proportion of bats showing titer >0.5 IU/ml in the region with a recent outbreak. However, low titers were still detected in bats from regions reporting the last outbreak of rabies at least 3 years prior to sampling. This study suggests that serological surveillance of rabies in vampire bats can be used as a tool to evaluate risk of outbreaks in at risk populations of cattle and human.

## Introduction

Rabies is an infectious disease of viral etiology that causes acute encephalitis, with rapid and usually fatal evolution in all mammals (1). The etiological agent is the RNA virus belonging to the family *Rhabdoviridae* and genus *Lyssavirus*, and it is a zoonosis distributed worldwide that now affects mainly low-income countries (2). Rabies virus is transmitted by contact with infected saliva through bites or scratches. Dogs are the main reservoir of rabies and are responsible for most fatal cases in humans worldwide (2). However, in Latin America, with success obtained by the countries to control rabies mediated by dogs through the mass vaccination campaign (3, 4), bats have become the main reservoir of rabies over the last decade and are responsible for thousands of cases in livestock and for most of the remaining cases in humans (1, 4). Within bats, the common vampire bat *Desmodus rotundus* is the most important reservoir because of the high occurrence of spillover of rabies from this species of bat to other animals and humans, and vampire bat rabies (VBR) remains unpredictable and uncontrolled in several areas of the continent.

By feeding every night on livestock, VBR causes significant economic losses in Latin America, particularly to small-scale farmers (5). *D. rotundus* can also feed on the blood of human beings, resulting in occasional and unpredictable outbreaks of VBR in remote settings such as the Amazon region (6). The epidemiology of VBR circulation among *D. rotundus* that results in rabies cases among livestock is driven by several natural factors that are still poorly understood. Anthropogenic and ecological features favor the presence of bats (e.g., distribution of cattle herd and land occupation) (7), and factors favoring VBR among bats are also involved. However, understanding VBR circulation requires the capacity of measuring rabies exposure or infections within the bat population.

Most studies understanding rabies circulation among bats rely on official reports of livestock mortality, but this data is often biased by variable levels of under-reporting across the landscap (8, 9). Herbivores are accidental hosts of the rabies virus. Because they are a dead-end host, they only contribute as sentinels in the existence of the virus in the bat population (7). Despite its limitations, studies based on livestock mortality have shown that rabies can circulate in the landscape in different ways including wave-like spread into new areas, metapopulation dynamics, or endemically (9–11). However, the dynamics of the virus in endemic areas where the virus has been established for longer periods of time is less understood. Therefore, surveillance of rabies in bats, although logistically challenging, can bring unique insides into the circulation of the virus and our ability to predict and prevent future outbreaks in humans and livestock (9, 11).

The recognition of bats as reservoirs of the disease made epidemiological surveillance extend to these species. Rabies virus is rarely isolated from an infected bat because infected bats are often lower than 1% of the population (11). Because the presence of anti-rabies antibodies correlates with exposure to the virus, serological studies can contribute to understanding VBR circulation among bats. Several studies have shown the presence of antibodies to rabies in bats that do not die from the disease, as seen in other animals and also in humans (12, 13), implying that exposure to rabies in this species does not necessarily leads to mortality (14). For example, in the Botucatu region of Brazil, an endemic region of VBR, 45% of *D. rotundus* vampire bats had virus neutralizing antibodies (VNA) titers ranging from 0.10 to 0.20 IU/ml, 9.31% had between 0.20 and 0.3 IU/ml, and 11% had VNA levels >0.30 IU/ml (14). Similarly, *D. rotundus* bats from 16 colonies were tested in the endemic region of Paraiba in Brazil and all animals presented anti-rabies antibodies titers in some level, with titers higher than 0.5 IU/ml in 30.1% of bats (15). Delpietro et al. (16) reported the detection of 30% antibodies in bats after an outbreak of bovine rabies in Argentina. Before the outbreak, 2% of the bats had the antibodies, and during the outbreak, the percentage increased to 4%, suggesting that the presence of high proportion of seropositivity results from a high circulation of the VBR among bats at the time of an outbreak.

Here, we took advantage of recent outbreaks of VBR among livestock in the Sao Paulo region of Brazil to further test whether seroprevalence in *D. rotundus* reflects the incidence of rabies in bat populations. Field work was combined with captive experiments to follow seropositive animals and increase our understanding of within-host anti-rabies antibodies dynamics among *D. rotundus*.

## Materials and Methods

### Virus Neutralizing Antibodies (VNA) in *D. rotundus* Bats During and After Outbreaks

**Figure 1:**
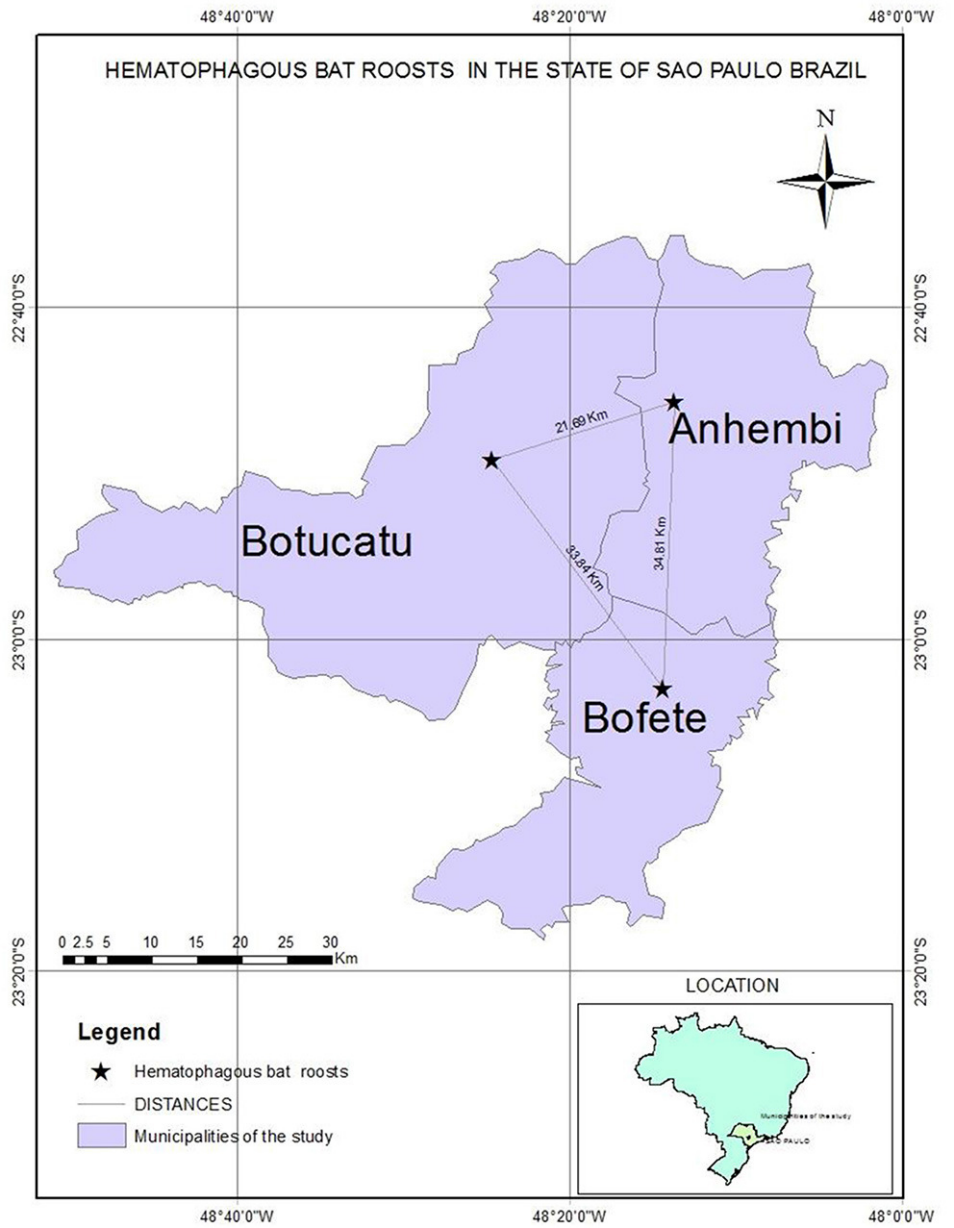
Map showing the three municipalities with different epidemiological scenarios.

We captured and collected samples of 64 *D. rotundus* from December 2015 to June 2016 from colonies in areas with three different epidemiological scenarios (Figure 1) of outbreaks in livestock of nearby farms attacked by these bats (according to the Ministry of Agriculture, Livestock and Supply from Brazil). Bats were aleatorily captured using nets installed outside the roost. In the first location, 23 bats were caught from roosts located in the University farm of UNESP, Botucatu, São Paulo. This farm just had a rabies outbreak 1 month before sampling involving seven cattle and horses. A second collection of five bats was performed 30 days after in this same spot. The second site was Bofete, where eight bats were captured and the nearby farms had reported rabies among livestock in 2013 and 2016. After 30 days, 16 bats were captured in the same farms. The third site of capture was Anhembi where six bats were captured and rabies has been reported on livestock in 2015 for the last time. After 30 days, six more bats were captured in the same place.

### Serum Sampling and Rabies Neutralizing Antibody Testing

From the second capture, six bats from Anhembi, four bats from Botucatu, and six from Bofete were aleatorily separated and kept in captivity to study their rabies antibodies for 4 months. The animals were quarantined under observation in isolated cages in a controlled environment in the animal facility of the infectious diseases sector—School of Veterinary Medicine and Animal Science-UNESP/Botucatu (approved by the Ethical Committee of Animals Uses in Veterinary Medicine and Animal Production of São Paulo State University “Julio de Mesquita Filho,” number 85/2015, and SISBIO (Biodiversity Authorization and Information System) license number 51231). Animals were fed with defibrinated bovine blood negative for anti-rabies antibodies using automatic drinking bottles, in addition to daily cleaning of the cages. In all bats captured in nature and not kept in captivity, blood samples were followed by euthanasia using halothane anesthetic induction and deepening of the anesthetic plane with 0.1 ml intraperitoneal ketamine. Bats kept in captivity were subjected to monthly blood collection with anesthetic induction. Blood was collected from the saphenous vein using needles 25 G ½ (0.45 × 13 mm) and capillaries (microhematocrit) with prior asepsis with 70% alcohol. Sera were stored at −20°C until antirabies neutralizing antibodies titer evaluation by the rapid fluorescent focus inhibition test (RFFIT) (17). For the RFFIT (18), a constant dose of a previously titrated (calibrated to give 80% fluorescent focus infected cells) cell culture-adapted RABV challenge virus (CVS) was incubated with 3-fold dilutions of the sera. After incubation of the serum-virus mixtures, a suspension of a clone of BHK-21 (BSR) cells was added. After 20 h of incubation, the cell monolayer was acetone-fixed and labeled with anti-rabies virus antibody conjugated to FITC (19). The optimal challenge dose (the dilution giving 80% infected cells for each virus production) was calculated. Titers of sera were calculated by comparison with a reference serum calibrated to the WHO reference serum. A minimal threshold of 0.5 IU/ml was considered for protection.

Animals that died during observation period (7) and those euthanized on the day of capture (or at the end of the 4 month period) were subjected to the same protocols of cardiac puncture for collection of blood used for RFFIT, and brain was submitted to qRT-PCR for rabies diagnosis as previously described (20), resulting all negatives to rabies.

Given the low number of samples from each type of colony, we used a Pearson's chi-squared test using the R function prop test to compare the proportion of seropositive individuals between day 0 and day 30 of sampling and between sampling locations.

## Results

### Virus Neutralizing Antibodies (VNA) During and After the Outbreak

**Figure 2:**
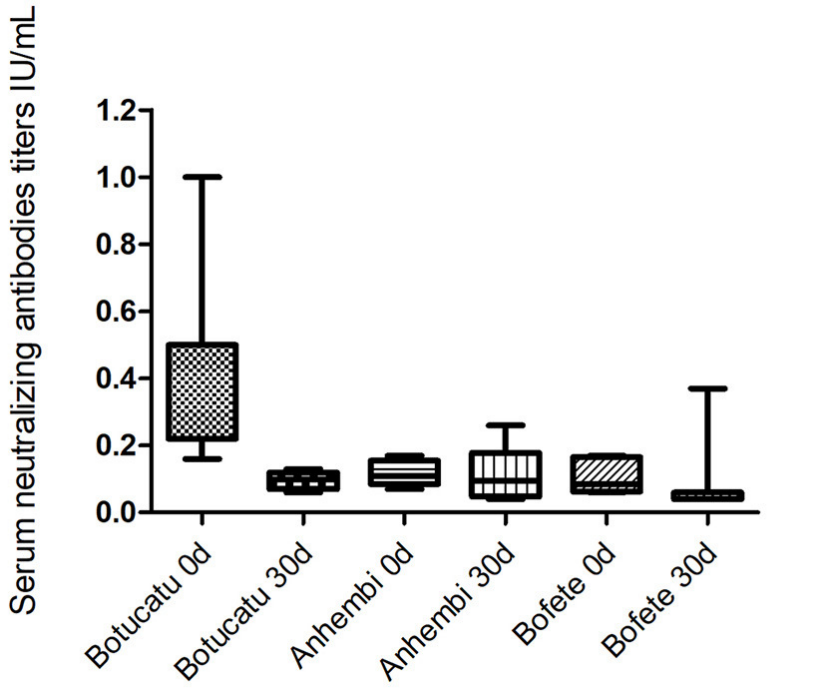
Geometric mean titers (GMT) of virus-neutralizing antibodies in the bats from the studied municipalities. Municipalities with different scenarios evaluated at 0 and 30 days.

The highest rabies antibodies titers were obtained from bats captured in Botucatu (0.45 ± 0.05), which had the most recent outbreaks. In this area, 13 animals out of 23 had protective antibody titers (0.64 ± 0.05). Average titer concentrations in the bats captured 30 days after (0.09 ± 0.01) decreased significantly (*p* = 0.0075), and none of those five bats had protective titers. Bats from Anhembi and Bofete had a similar average concentration of titers (0.11 ± 0.01 and 0.10 ± 0.01; *p* = 0.51), respectively, that were lower than in Botucatu (0.45 ± 0.05) at the first capture (*p* < 0.0001). However, bats from Anhembi maintained the same level of titer 30 days later (0.11 ± 0.03), whereas Bofete presented a titer >10 IU/ml, which increased the overall average concentration (0.70 ± 0.63) (Figure 2). Considering that this bat could interfere with the biological interpretation of our results, we run another statistics test excluding its titer (>10 IU/ml) to the analysis. The statistical analysis showed a significant difference of the VNA titers from bats captured in Bofete in both moments (*p* = 0.0129). Overall, the proportion of seropositive animals, on day 0, was higher in Botucatu compared to either Anhembi and Bofete (Pearson's test, *p* < 0.01), and there was no significant difference in the proportion of seropositive individuals between day 0 and 30 in neither of three locations (Pearson's test, *p* > 0.05). All captured bats were apparently in a good sanitary condition, and no clinical signs were observed. Details on specific VNA are given in Table 1, Figure 2.

**Table 1:** Antirabies neutralizing antibodies titers in *Desmodus rotundus* bats captured, from December 2015 to june 2016, in geographic areas with different epidemiological situation for rabies among livestock.

***Desmodus rotundus*** **bats**	**Botucatu 0 d**	**Botucatu 30 d**	**Anhembi 0 d**	**Anhembi 30 d**	**Bofete 0 d**	**Bofete 30 d**
1	0.50	0.13	0.17	0.13	0.06	0.06
2	1.00	0.06	0.07	0.26	0.15	0.04
3	0.22	0.11	0.12	0.15	0.17	0.04
4	0.66	0.08	0.09	0.04	0.07	0.12
5	0.25	0.10	0.10	0.06	0.08	0.04
6	0.33		0.15	0.05	0.06	0.37
7	0.66				0.09	0.04
8	1.00				0,17	0.04
9	0.50					0.06
10	0.50					0.04
11	1.00					0.04
12	0.22					0.04
13	0.16					0.04
14	0.16					0.05
15	0.50					0.04
16	0.22					10.22
17	0.50					
18	0.50					
19	0.50					
20	0.22					
21	0.50					
22	0.16					
23	0.16					
**Median ± sd**	0.45 ± 0.05	0.09 ± 0.01	0.11 ± 0.01	0.11 ± 0.03	0.10 ± 0.01	^*^0.07 ± 0.02
**Seropositive/total bats captured**	**13/23**	**0/5**	**0/6**	**0/6**	**0/8**	**1/16**
**Cattle outbreaks in nearby farms**	**Recent outbreak of rabies (1 month before)**		**Rabies reported in 2015**		**Rabies reported in 2013 and 2016**	

* *Median ± sd excluding the bat with titer 10.22. Bold values represents collection not performed due to technical difficulty or death of animals*.

### Virus Neutralizing Antibodies (VNA) Profile of Bats Kept in Captivity

**Figure 3:**
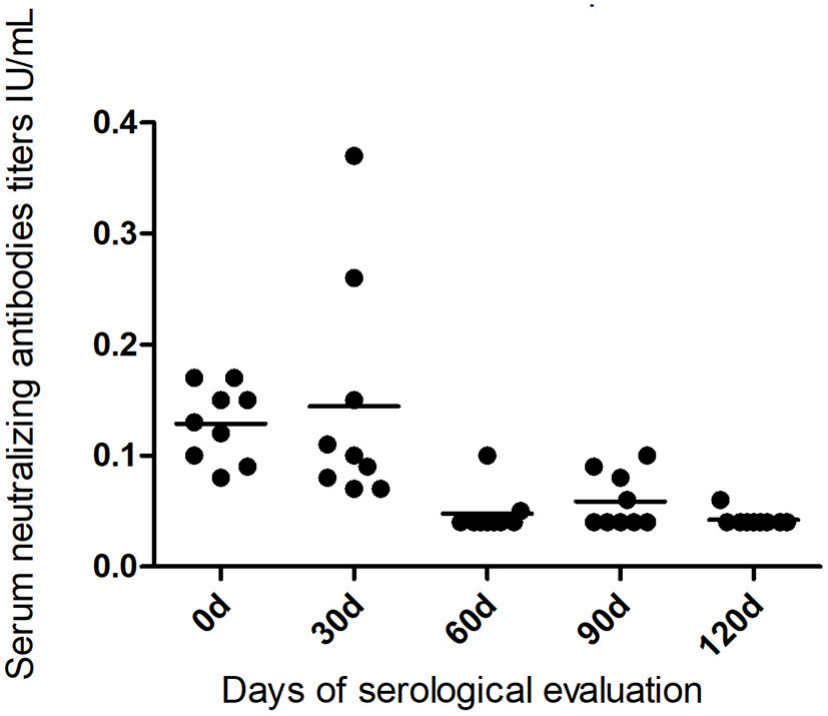
Serological profile of virus-neutralizing antibodies in the bats kept in captivity for 120 days from the studied municipalities.

During the observation period, seven bats died and resulted negative by rabies virus qRT-PCR test, so it was possible to have the serological profile from nine bats during the 120 days, one bat from Botucatu, and four bats from Bofete and Anhembi, respectively. The serological profile of bats kept in captivity showed a constant decrease in the titer levels in all animals (Table 2, Figure 3) up to days 120, where all titers were lower than 0.10 (*p* < 0.0001). The statistical analysis of the VNA titers from day 0 compared to 60 days showed a significant difference (*p* = 0.0009), and the same result was obtained when comparing to 90 days (*p* = 0.0017) and 120 days (*p* = 0.0004). Statistical significance was also evidenced comparing 30–60 days (*p* = 0.0017), 90 days (*p* = 0.0090), and 120 days (*p* = 0.0004), demonstrating a decrease in the levels of VNA in bats in captivity. Although two bats showed an increase in the VNA at 30 days, it is not possible to make any conclusion due to the low number of animals studied.

**Table 2:** Antirabies antibodies titers from bats captured in regions with different epidemiological situation.

**Bat**	**Origin**	**0 d**	**30 d**	**60 d**	**90 d**	**120 d**
1	Botucatu	0.13	0.09	0.04	0.04	0.04
2	Anhembi	0.17	0.07	0.05	0.08	0.04
3	Anhembi	0.12	0,11	0,04	0,1	0,04
4	Anhembi	0.1	0.26	0.04	0.04	0.04
5	Anhembi	0.15	0.08	0.04	0.04	0.04
6	Bofete	0.15	0.37	0.1	0.09	0.06
7	Bofete	0.17	0.15	0.04	0.04	0.04
8	Bofete	0.08	0.07	0.04	0.04	0.04
9	Bofete	0.09	0.1	0.04	0.06	0.04
Median ± sd		0,12 ± 0.01	0.14 ± 0.02	0.04 ± 0.019	0.05 ± 0.02	0.04 ± 0.02

*Botucatu: Recent outbreak of rabies (1 month before); Anhembi: Rabies reported in 2015; Bofete: Rabies reported in 2013 and 2016*.

## Discussion

Serological studies of VBR comparing populations of bats with different scenarios of rabies outbreaks in cattle are rare but crucial to understand within-bat dynamics of rabies. We found that vampire bats from the region of Botucatu, which had experienced a recent outbreak of rabies in cattle 30 days prior to sampling, showed a high proportion of seropositive individuals compared to two other sampling sites where the latest outbreak in cattle was reported at least 3 years prior to sampling. In Botucatu, 30 days after the outbreak, half of the bats had antibodies considered to be protective against rabies, which decreased in proportion 60 days after the outbreak in the region. Seropositive animals kept in captivity showed a variable decreased in titer after 4 months, suggesting a rapid loss of antibodies to non-protective levels. Our study suggests that rabies serology in bats can be an accurate indicator to identify if rabies had circulated in bats sampled.

The high proportion of antibodies considered as protective for rabies among bats sampled in Botucatu suggests the presence and circulation of the virus (8, 11) and corroborates cases reported among livestock in nearby farms. Although the second collection of bats 30 days later was much smaller (*N* = 5), there was a decrease on the proportion of seropositive animals. This decrease suggests that not all bats in a colony are exposed to the virus and that rabies does not necessarily persist within a colony after an outbreak (16).

Although outbreaks from cattle in Bofete and Anhembi were not reported for at least 3 years, antibodies were detected among bats in the first capture in both colonies, but not high enough to be considered protective (<0.5 IU/ml). This could reflect recent exposure to low levels of the virus that does not result in disease symptoms including exposure shortly after birth, subclinical or asymptomatic infections, sublethal infection, carrier state, or latent virus activated by stress (11, 21–23). In fact, Blackwood et al. (11) suggested that the probability of developing a lethal infection upon exposure to rabies is low in vampire bats (~10%), which enables viral persistence in the slowly reproducing bat colonies. This is also observed in other bats species (14, 24).

Low levels of antibodies within a bat colony without recent exposure could also result from long-term persistence of antibodies if rabies exposure happened several years in that colony (8, 25). However, the duration of rabies antibodies after exposure without re-exposure remains poorly understood for vampire bats. In this study, anti-rabies antibodies decreased to low levels 60 days after capture of seropositive individuals but fluctuations and sudden increases were also observed in some individuals. Antibodies fluctuation has been reported in free bats but explained by re-exposure to the virus, which was not possible in our laboratory settings (26). This result suggests possible rabies virus persistence in the bat tissue with periodic antigenic stimulation. This calls for future studies on the mechanisms behind anti-rabies antibody production and on the immunological and protective consequences of having low levels of antibodies in healthy bats.

The increase of anti-rabies antibodies titers on the second capture observed in Bofete suggests that rabies circulation can be detected in the bat population despite cases not been reported among livestock. This highlights the possibility of using bat serology surveillance to predict risk in that population and therefore affects livestock in the region too. Therefore, cost-effective analysis and more understanding on the serological behavior of bat population are needed to evaluate whether rabies serological surveillance among bats can be added as a tool to improve our understanding and prevention of rabies risk in both livestock and humans.

Overall, our study shows that anti-rabies antibodies among vampire bats could reflect the current risk of rabies outbreaks among nearby cattle. Furthermore, we observed a decrease in antibody titers 2 month after animals had high titers, although titers fluctuated in time and between individual bats. Future studies are required to assess whether the observed titer levels >0.5 IU/ml are indeed protective against rabies on bats and whether low-titers can also reflect rabies circulation among bats and subsequent risk to livestock and humans.

## Data Availability Statement

The raw data supporting the conclusions of this article will be made available by the authors, without undue reservation.

## Ethics Statement

The animal study was reviewed and approved by CEUA FMVZ.

## Author Contributions

FC, LB, BR, CA, and JM Bats capture, blood collection and work execution. JB, JM, and LB Foreground research, writing and editing. IK, KS, SS, AR, GB, and LA Serology. All authors contributed to the article and approved the submitted version.

## Conflict of Interest

The authors declare that the research was conducted in the absence of any commercial or financial relationships that could be construed as a potential conflict of interest.

## Publisher's Note

All claims expressed in this article are solely those of the authors and do not necessarily represent those of their affiliated organizations, or those of the publisher, the editors and the reviewers. Any product that may be evaluated in this article, or claim that may be made by its manufacturer, is not guaranteed or endorsed by the publisher.
