# Supplementary material for: Serological Surveillance of Rabies in Free-Range and Captive Common Vampire Bats Desmodus rotundus
Source: Front Vet Sci. 2021 Sep 29;8:681423. doi: 10.3389/fvets.2021.681423 (PMC8511519; doi:10.3389/fvets.2021.681423)
Supplement: Supplementary file 1 [file Data_Sheet_1.PDF]

Municipality	Day 0 (seropositive/total)	Day 30	Cattle outbreaks in nearby farms
Botucatu	0.4530 ± 0.05655 (n=13/23)	0.0960 ± 0.01208 (n=0/5)	Recent outbreak rabies (1 month before)
Anhembi	0.1167 ± 0.01542 (n=0/6)	0.1150 ± 0.03433 (n=0/6)	Rabies reported in 2015
Bofete	0.1063 ± 0.01721 (n=0/8)	0.7050 ± 0.6347 (n=1/16)	Rabies reported in 2013

Table 1. Anti-rabies neutralizing antibodies in Desmodus rotundus bats captured in municipalities with different epidemiological situation for rabies among livestock. Data are summarized as median± sd, with n=seropositive (>0.5IU/ml) /total capture animals.
