# Supplementary figures and images for: Serological Surveillance of Rabies in Free-Range and Captive Common Vampire Bats Desmodus rotundus
Source: Front Vet Sci. 2021 Sep 29;8:681423. doi: 10.3389/fvets.2021.681423 (PMC8511519; doi:10.3389/fvets.2021.681423)

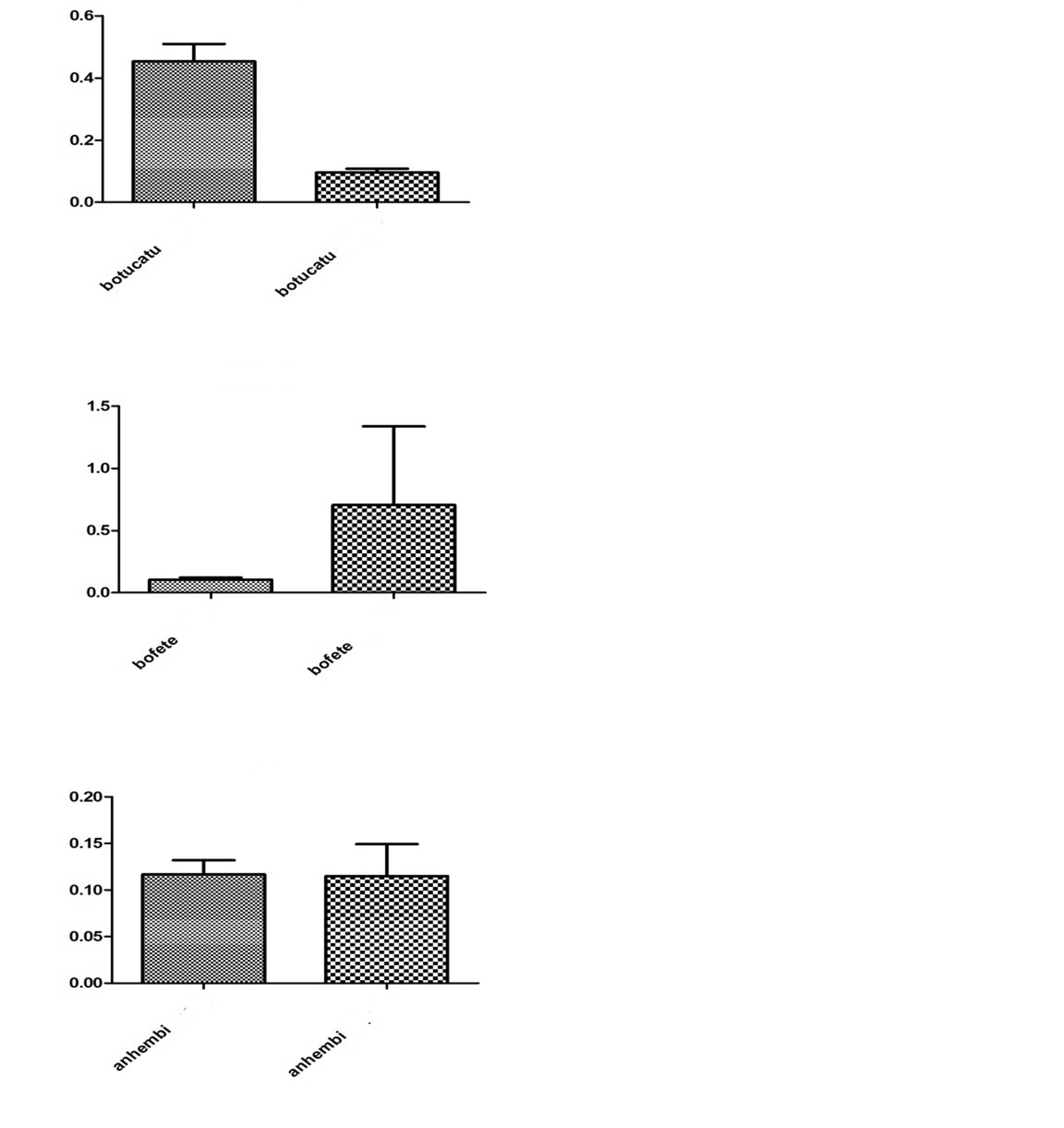

Supplement: Supplementary file 2 [file Image_1.JPEG]
